# p^13^CMFA: Parsimonious ^13^C metabolic flux analysis

**DOI:** 10.1371/journal.pcbi.1007310

**Published:** 2019-09-06

**Authors:** Carles Foguet, Anusha Jayaraman, Silvia Marin, Vitaly A. Selivanov, Pablo Moreno, Ramon Messeguer, Pedro de Atauri, Marta Cascante

**Affiliations:** 1 Department of Biochemistry and Molecular Biomedicine & Institute of Biomedicine of University of Barcelona, Faculty of Biology, Universitat de Barcelona, Barcelona, Spain; 2 Centro de Investigación Biomédica en Red de Enfermedades Hepáticas y Digestivas (CIBEREHD) and Metabolomics node at Spanish National Bioinformatics Institute (INB-ISCIII-ES-ELIXIR), Instituto de Salud Carlos III (ISCIII), Madrid, Spain; 3 European Molecular Biology Laboratory, European Bioinformatics Institute (EMBL-EBI), Cambridge, United Kingdom; 4 LEITAT Technological Center, Health & Biomedicine Unit, Barcelona, Spain; Ecole Polytechnique Fédérale de Lausanne, SWITZERLAND

## Abstract

Deciphering the mechanisms of regulation of metabolic networks subjected to perturbations, including disease states and drug-induced stress, relies on tracing metabolic fluxes. One of the most informative data to predict metabolic fluxes are ^13^C based metabolomics, which provide information about how carbons are redistributed along central carbon metabolism. Such data can be integrated using ^13^C Metabolic Flux Analysis (^13^C MFA) to provide quantitative metabolic maps of flux distributions. However, ^13^C MFA might be unable to reduce the solution space towards a unique solution either in large metabolic networks or when small sets of measurements are integrated. Here we present parsimonious ^13^C MFA (p^13^CMFA), an approach that runs a secondary optimization in the ^13^C MFA solution space to identify the solution that minimizes the total reaction flux. Furthermore, flux minimization can be weighted by gene expression measurements allowing seamless integration of gene expression data with ^13^C data. As proof of concept, we demonstrate how p^13^CMFA can be used to estimate intracellular flux distributions from ^13^C measurements and transcriptomics data. We have implemented p^13^CMFA in Iso2Flux, our in-house developed isotopic steady-state ^13^C MFA software. The source code is freely available on GitHub (https://github.com/cfoguet/iso2flux/releases/tag/0.7.2).

## Author summary

^13^C Metabolic Flux Analysis (^13^C MFA) is a well-established technique that has proven to be a valuable tool in quantifying the metabolic flux profile of central carbon metabolism. When a biological system is incubated with a ^13^C-labeled substrate, ^13^C propagates to metabolites throughout the metabolic network in a flux and pathway-dependent manner. ^13^C MFA integrates measurements of ^13^C enrichment in metabolites to identify the flux distributions consistent with the measured ^13^C propagation. However, there is often a range of flux values that can lead to the observed ^13^C distribution. Indeed, either when the metabolic network is large or a small set of measurements are integrated, the range of valid solutions can be too wide to accurately estimate part of the underlying flux distribution. Here we propose to use flux minimization to select the best flux solution in the^13^C MFA solution space. Furthermore, this approach can integrate gene expression data to give greater weight to the minimization of fluxes through enzymes with low gene expression evidence in order to ensure that the selected solution is biologically relevant. The concept of using flux minimization to select the best solution is widely used in flux balance analysis, but it had never been applied in the framework of ^13^C MFA. We have termed this new approach parsimonious ^13^C MFA (p^13^CMFA).

## Introduction

Fluxomics is the omics field that analyses metabolic fluxes (i.e., reaction and transport rates in living cells) which are a close reflection of the metabolic phenotype. As such, quantitative tracking of metabolic fluxes is vital for deciphering the regulation mechanisms of metabolic networks subjected to perturbations, including disease states and drug-induced stress. However, unlike other omics data that can be quantified directly, the fluxome can only be estimated through an indirect interpretation of experimental data[1–3].

There are two main model-based approaches to quantifying metabolic fluxes, Flux Balance Analysis (FBA) and ^13^C Metabolic Flux Analysis (^13^C MFA). Both methods use stoichiometric, thermodynamic and experimental constraints to find the range of feasible fluxes across a metabolic network and then find the flux distributions within that space that optimize a given objective function. However, both techniques differ in the type of objective function optimized.

In FBA, the objective function is a set of fluxes to be minimized or maximized. These fluxes must represent a biological objective deemed desirable in the conditions of study (e.g., synthesis of biomass components for proliferating systems)[4]. A significant limitation of FBA is that the choice of objective(s) can significantly influence the predicted flux distributions.

In ^13^C MFA, the objective function is to minimize the difference between simulated and measured ^13^C enrichment in metabolites [5,6]. ^13^C enrichment is quantified in metabolic products and intermediates after incubating samples with metabolic substrates labeled with ^13^C (tracers) and provides information about how carbons are redistributed along metabolic pathways[7]. Compared to FBA, ^13^C MFA has a greater capacity to elucidate the fluxes of central carbon metabolism. However, ^13^C MFA is more complex to solve than FBA due to the non-linear nature of the ^13^C MFA objective.

A significant limitation of FBA is that there is generally a wide range of optimal flux distributions[8]. This is not usually the case with ^13^C MFA which can generally determine flux distributions with a high degree of accuracy. ^13^C MFA achieves this by integrating large sets of measured isotopologue fractions from parallel experiments with tracers optimized for different parts of the network[9–16]. However, when ^13^C MFA is used in large metabolic networks and with a limited set of measurements, it can also suffer from the same limitation as FBA and result on a wide interval of flux values for part of the metabolic network[5,17–19].

On FBA, an approach to reduce the range of optimal solutions consists in running a second optimization step on the optimal solution range. One of such methods is parsimonious FBA (pFBA)[20]. This approach, which follows the principle of parsimony or simplicity, consists on finding the optimal value of the primary objective function through FBA and then running a second optimization step where the sum of reaction fluxes is minimized while maintaining the optimal primary objective. The GIMME (and its derivative GIM^3^E) algorithms[21,22] are based on a similar principle as pFBA. Unlike standard pFBA, where all reactions fluxes are minimized with equal weight, GIMME integrates gene expression data to give greater weight to the minimization of fluxes through reactions catalyzed by lowly expressed enzymes. Different to FBA, for ^13^C MFA, there is currently no approach that relies on a second optimization to reduce the solution space when experimental data is insufficient to constrain the system towards a unique solution.

In addition to model-based approaches (e.g., FBA or ^13^C MFA), metabolic fluxes can also be analyzed through the direct or semidirect interpretation of ^13^C data. This approach primarily consists of predicting the contribution of a labeled substrate to the synthesis of a given metabolite (nutrient contribution) and predicting the relative activity of pathways (pathway activity analysis). Pathway activity analysis assumes that the isotopologue fractions used as a surrogate for the pathways of interest are primarily generated through them. This assumption is generally based on the assertion that the pathways of interest are the most direct way to generate such fractions from the labeled substrate used in the experiment[2,7,23–25]. Unlike ^13^C MFA, direct interpretation of ^13^C data is generally not able to quantify network-wide flux maps. Instead, it provides a series of qualitative or semiquantitative flux predictions around each analyzed metabolite. Strategies that couple direct interpretation of ^13^C data to regression and correlation analyses are widely applied to unveil the effect of an external perturbation, such as a therapeutic intervention, on central carbon metabolism[26–30].

**Fig 1:**
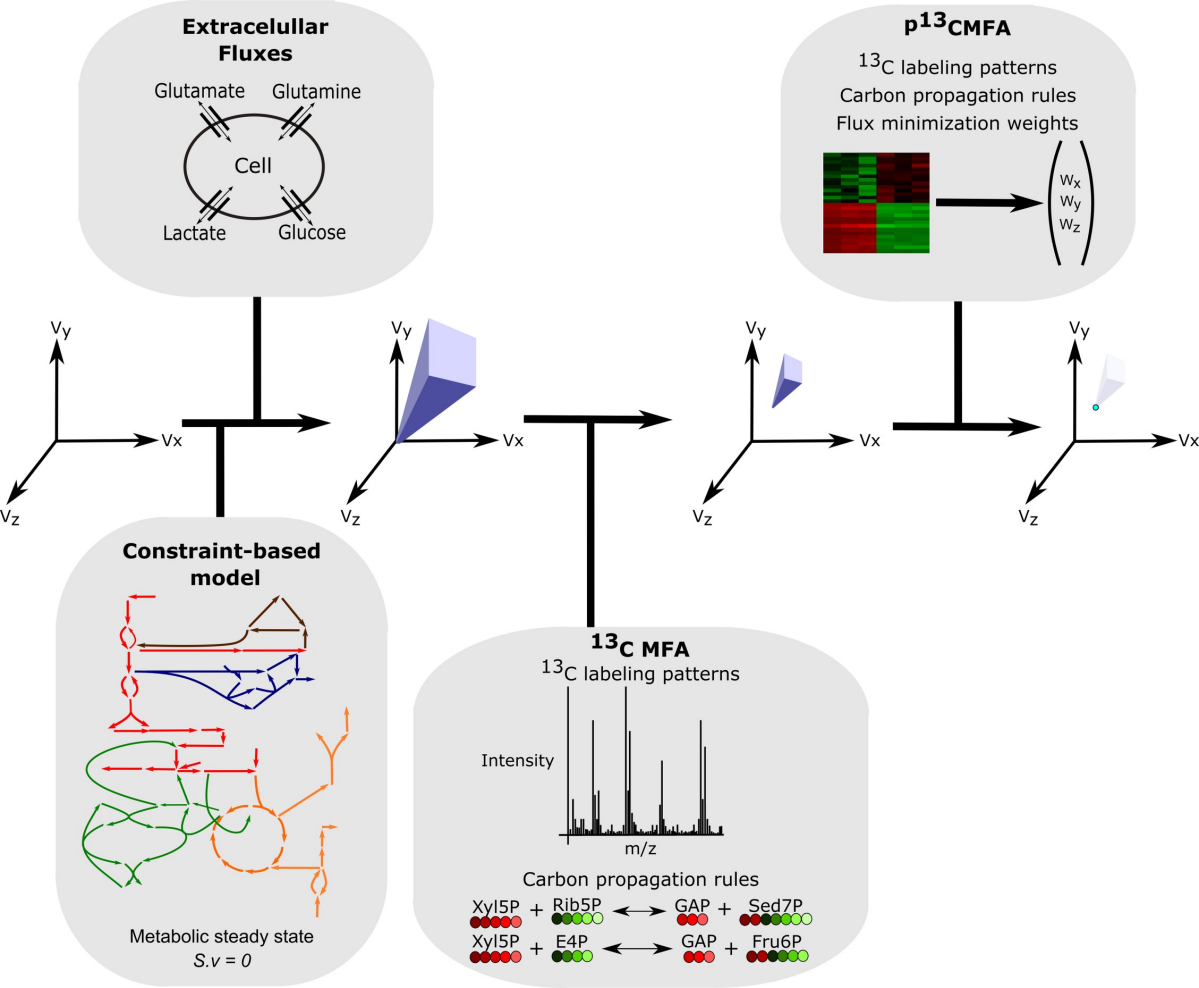
The conceptual basis of p^13^CMFA. From an infinite space of flux (*v*) solutions, a space of feasible solutions is obtained through the integration of stoichiometric and thermodynamic constraints (in the form of a constraint-based model) and the measured extracellular fluxes. Applying ^13^C MFA to integrate experimental ^13^C data can further reduce the solution space to those flux distributions that are consistent with such data. Through flux minimization, p^13^CMFA can identify the optimal flux distribution that lies on the edge of the ^13^C MFA solution space. Such minimization can be weighted according to the gene expression evidence for each enzyme.

Here we present parsimonious ^13^C MFA (p^13^CMFA), a new model-based approach to flux estimation. p^13^CMFA first minimizes the difference between experimental and simulated ^13^C enrichment in metabolites (^13^C MFA) and then applies the flux minimization principle to select the best solution among the solutions that fit experimental ^13^C data. Hence, p^13^CMFA can be used to select the best flux map in instances where experimental ^13^C measurements are not enough to fully constrain the ^13^C MFA solution space. Furthermore, the minimization can be weighted by gene expression allowing seamless integration of ^13^C with gene expression data (Fig 1).

We have implemented p^13^CMFA in Iso2Flux, our in-house developed isotopic steady-state ^13^C MFA software (https://github.com/cfoguet/iso2flux/releases/tag/0.7.2). As a proof of concept, we have applied it to the analysis of the metabolic flux distribution in HUVECs (Human umbilical vein endothelial cells) through the integration of a small set of ^13^C enrichment measurements and transcriptomics data. Furthermore, we validated the predictive capacity of p^13^CMFA using data from a published study of HTC116 cells where fluxes had been estimated with a high degree of confidence[14]. Using only a small subset of the measurements from such study, p^13^CMFA was able to achieve significantly better flux predictions than both ^13^C MFA and GIMME.

## Results

### Description of the p^13^CMFA approach

p^13^CMFA consists of two consecutive optimizations: first, the optimal solution to the ^13^C MFA problem is identified (Eq 1); secondly, the weighted sum of reaction fluxes is minimized within the optimal solution space of ^13^C MFA (Eq 2).

The ^13^C MFA optimization (Eq 1) identifies the flux distribution that minimizes the difference between measured and simulated isotopologue fractions [5,7]:

$$X_{opt}=min\sum_{j}{\left(\frac{E_{j}-Y_{j}(v)}{\sigma_{j}}\right)}^{2}$$ (Eq 1)

SubjecttoS.v=0,lb≤v≤ub

where,

*v* is a vector of flux values describing a valid steady-state flux distribution;

*X*_*opt*_ is the optimal value of the ^13^C MFA objective;

*E*_*j*_ is the experimentally quantified fraction for isotopologue *j*;

*Y*_*j*_*(v)* is the simulated isotopologue fraction for isotopologue *j* with flux distribution *v*. Such simulation is performed by solving a complex non-linear system of equations built around isotopologues balances [1].

*σ*_*j*_ is the experimental standard deviation of the measurements of isotopologue *j*;

*S* is the stoichiometric matrix;

*lb* and *ub* are vectors defining the upper and lower bounds for flux values. Flux bounds can be used to integrate experimental flux measurements;

Either in large metabolic networks or when small sets of ^13^C measurements are integrated, the ^13^C MFA problem can be undetermined and there can be a wide range of possible solutions. Such indetermination emerges from cycles and alternative pathways in the metabolic network, which lead to many possible flux combinations that can result in the measured ^13^C label patterns. Furthermore, many of the ^13^C MFA solutions can involve large fluxes through futile cycles, which are usually artifacts of the optimization process as *in vivo* enzyme activities cannot support such large flux values. Therefore, to select the best solution among the many solutions that fit experimental ^13^C data, p^13^CMFA runs a second optimization where the weighted sum of fluxes is minimized (Eq 2):

$$min\sum_{i}|v_{i}|\cdot w_{i}$$ (Eq 2)

$$subject\;to\;S.v=0,\;lb\leq v\leq ub,\;\sum_{j}{\left(\frac{E_{j}-Y_{j}(v)}{\sigma_{j}}\right)}^{2}\leq X_{opt}+T$$

where:

*w*_*i*_ is the weight given to the minimization of flux through reaction *i*;

*T* is the maximum value that the ^13^C MFA objective can deviate from its optimal value (primary objective tolerance) when fluxes are minimized;

The difference between the optimal ^13^C MFA objective function value and the objective function value when the total reaction flux is minimized can be assumed to follow a Chi^2^-distribution with one degree of freedom. Therefore, setting T to 3.84 gives a p^13^CMFA solution within the 95% confidence intervals of ^13^C MFA[5].

With p^13^CMFA, the activity through cycles is minimized to the minimum amount needed to account for experimental measurements. Furthermore, gene expression measurements can be integrated to give greater weight to the minimization of fluxes through reactions catalyzed by lowly expressed enzymes. Then, in instances where multiple pathways can result in similar label patterns, those pathways with stronger gene expression evidence are selected. Hence, p^13^CMFA reduces the solution space towards a unique solution without requiring a simplification of the metabolic network or additional ^13^C measurements (Fig 1).

### Example of p^13^CMFA usage

As an example of a potential application of p^13^CMFA, we applied it to analyze the metabolic flux distribution in HUVECs using a publicly available dataset not large enough to make meaningful flux predictions with conventional ^13^C MFA.

In this study, available in the MetaboLights repository[31] (accession number MTBLS412), HUVECs were incubated in the presence of the tracer [1,2-^13^C_2_]-glucose, and the relative abundance of ^13^C isotopologues was quantified in glycogen, ribose, lactate, and glutamate. The rates of production/consumption of glucose, glycogen, lactate, glutamate, and glutamine were also quantified. The data were integrated into a stoichiometric model of central metabolism which includes glycolysis, glycogen metabolism, pentose phosphate pathway (PPP), tricarboxylic acid (TCA) cycle, fatty acid synthesis, and energy and redox metabolism (S1 ZIP).

**Fig 2:**
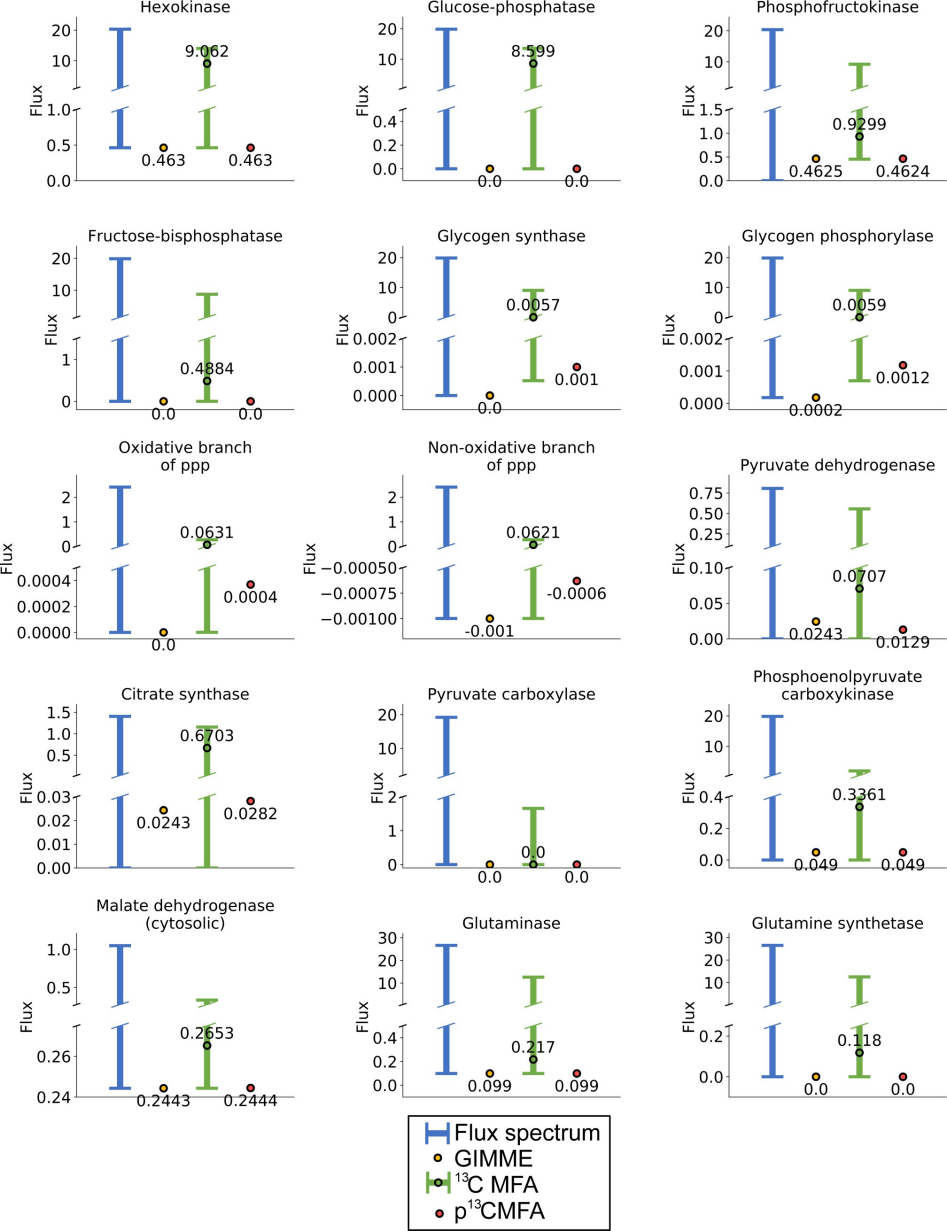
Flux spectrum, GIMME solutions, ^13^C MFA flux ranges, and p^13^CMFA solutions for some key reaction fluxes in the HUVECs case study. Flux spectrum represents the feasible flux ranges considering only the stoichiometric and thermodynamic constraints and the measured extracellular fluxes. GIMME flux values are obtained when total reaction flux is minimized weighted by gene expression without integrating ^13^C data. For ^13^C MFA, the flux values obtained after the ^13^C MFA optimization and the range of the 95% confidence intervals for such values are shown. The p^13^CMFA flux values are obtained when total reaction flux is minimized within the ^13^C MFA solution space. Fluxes are expressed in μmol·h^-1^·million-cells^-1^.

To predict the flux distribution using conventional ^13^C MFA, 95% confidence intervals were computed for each predicted flux value. From this analysis, the space of flux solutions consistent with the measured ^13^C enrichment was estimated. The resulting space of solution was still mostly undetermined and, in general, ^13^C MFA was unable to significantly constraint the flux ranges emerging from the stoichiometric and thermodynamic constraints and the measured extracellular fluxes (Fig 2, S1 Table). For instance, despite integrating measurements of ^13^C enrichment in ribose, it was not possible to conclude whether the oxidative branch of the pentose phosphate pathway contributed more to *de novo* ribose synthesis than the non-oxidative branch or vice versa.

**Fig 3:**
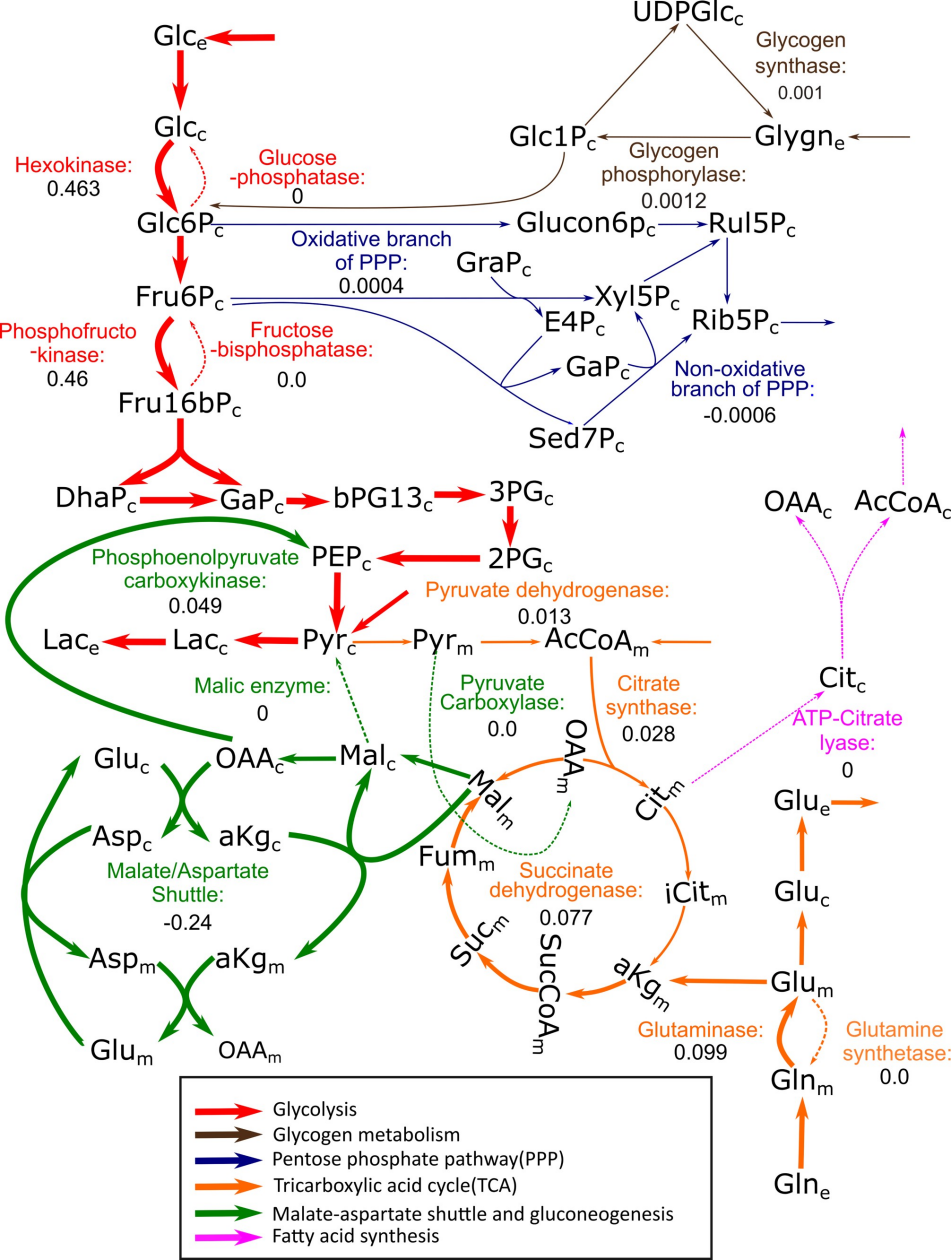
Schematic representation of the central carbon metabolism flux map obtained with p^13^CMFA in the HUVECs case study. Reaction fluxes are indicated for some key reactions in μmol·h^-1^·million-cells^-1^. Arrows indicate net flux direction, and line width is representative of flux magnitude. Reactions and metabolites of redox and energy metabolism have been omitted from this figure for clarity. 2PG: 2-Phosphoglycerate. 3PG: 3-Phosphoglycerate. AcCoA: Acetyl-CoA. aKG: α-Ketoglutarate. Asp: Aspartate. bPG13: 1,3-Bisphosphoglycerate. Cit: Citrate. DhaP: Dihydroxyacetone phosphate. Fru16bP: Fructose 1,6-bisphosphate. Fru6P: Fructose 6-phosphate. Fum: Fumarate. GaP: Glyceraldehyde-3-Phosphate. Glc: Glucose. Glc1P: Glucose 1-phosphate. Glc6P: Glucose 6-phosphate. Gln: Glutamine. Glu: Glutamate. Glucon6P: Gluconate 6-phosphate. Glygn: Glycogen. iCit: Isocitrate. Lac: Lactate. Mal: Malate. OAA: Oxaloacetate. PEP: Phosphoenolpyruvate. Pyr: Pyruvate. Rib5P: Ribose 5-phosphate. Rul5P: Ribulose 5-phosphate. Sed7P: Sedoheptulose 7-phosphate. Suc: Succinate. SucCoa: Succinyl-CoA. UDPGlc: Uridine diphosphate glucose. The subscripts *e*, *c*, and *m* denote the extracellular, cytosolic and mitochondrial compartments, respectively.

Nevertheless, p^13^CMFA can be applied to select the best solution in the ^13^C MFA solution space. With this aim, transcriptomic data taken from the literature[32] were used to add additional penalties to the flux through lowly expressed enzymes. Indeed, by applying p^13^CMFA, we can now conclude that, under the condition of the study, glucose is mostly directed towards lactate production except for a small part going to the TCA cycle through pyruvate dehydrogenase (Fig 2, Fig 3). Glutamine is mainly metabolized to glutamate or directed to glycolysis through the TCA cycle and phosphoenolpyruvate carboxykinase. In the PPP, the non-oxidative branch contributes to roughly 60% of the net ribose synthesis. Only the glycogen phosphorylase/glycogen synthase futile cycle is predicted to be active, while the remaining futile cycles (i.e., the hexokinase/glucose 6-phosphatase, phosphofructokinase/fructose bis-phosphatase, pyruvate carboxylase/phosphoenolpyruvate carboxykinase, and glutaminase/glutamine synthase cycles) are predicted to be inactive. Concerning redox metabolism, most of the reduced NAD+ (NADH) produced in the mitochondria is exported to the cytosol through the malate-aspartate shuttle, where it is used to reduce pyruvate to lactate.

To evaluate the contribution of ^13^C MFA to the p^13^CMFA solution, GIMME (i.e., flux minimization weighted by gene expression without integrating ^13^C data) was also performed (Fig 2, S1 Table). Lacking ^13^C data, GIMME does not predict any activity in the oxidative branch of the pentose phosphate pathway, nor on the glycogen phosphorylase/glycogen synthase futile cycle. Furthermore, GIMME predicts a significantly larger flux through pyruvate dehydrogenase than p^13^CMFA. Interestingly, p^13^CMFA predicts an increased activity of the TCA cycle compared to the GIMME solution. This increased activity is fueled by alternative sources of acetyl-CoA such as fatty acid oxidation or catabolism of ketogenic amino acids. Hence, p^13^CMFA is able to take advantage of measured ^13^C enrichments and predict significantly different flux maps than those derived from flux minimization alone.

### Validation of the p^13^CMFA approach

To validate the p^13^CMFA method, we used data from a metabolic characterization of the colon cancer cell line HCT 116 published by Tarrado-Castellarnau *et al*. [14]. In this study, 25 direct flux measurements and 24 sets of isotopologue fractions, measured after incubation with either [1,2-^13^C_2_]-glucose or [U-^13^C_5_]-glutamine, had been integrated in the framework of ^13^C MFA. With such a large set of experimental measurements, ^13^C MFA had been able to estimate the flux through 62 reactions with a high degree of accuracy. In the same study, transcriptomics data were also collected.

**Fig 4:**
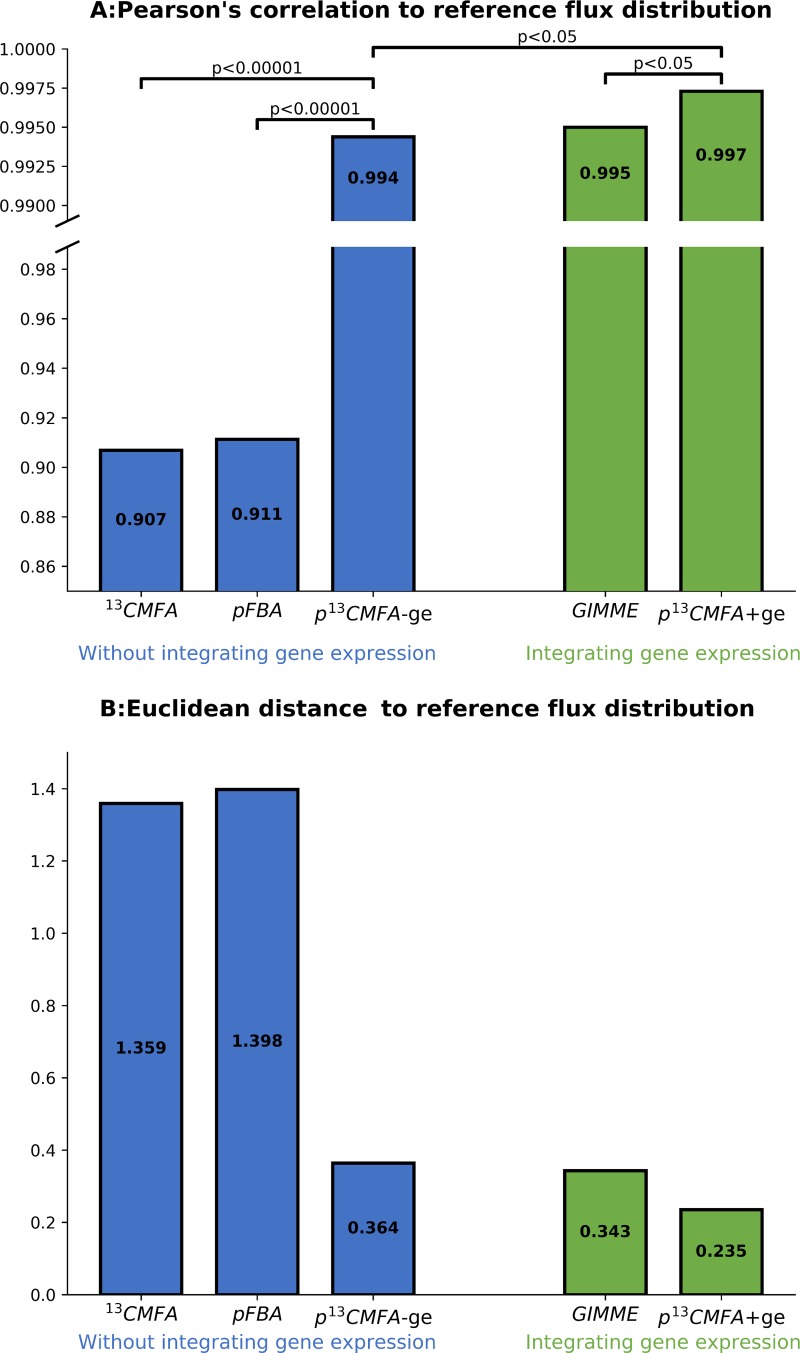
Comparison of the predictive power of ^13^C MFA, pFBA, GIMME, and p^13^CMFA. A: Pearson’s correlation coefficients between the reference flux distribution and the flux maps obtained from ^13^C MFA (optimal solution), pFBA, GIMME, and p^13^CMFA. p^13^CMFA was applied both with and without integrating gene expression data (p^13^CMFA+ge and p^13^CMFA-ge, respectively). The statistical significance of the difference between correlation coefficients was evaluated using the Fisher r-to-z transformation[33]. B: Euclidian distances between the reference flux distribution and the flux maps obtained from ^13^C MFA (optimal solution), pFBA, GIMME, and p^13^CMFA.

From this large data set, we selected a partial data set consisting of 7 experimental flux measurements (the rates of uptake/secretion of glucose, lactate, glutamine, glutamate and, oxygen and the rate of protein and glycogen synthesis) and 4 sets of isotopologue fractions (isotopologue fractions in ribose, lactate, glutamate and glycogen measured after incubation with 1,2-^13^C_2_]-glucose). Those are the sets of isotopologues and fluxes that were analyzed in the HUVECs case study with the addition of the rate of protein synthesis and oxygen consumption which Tarrado-Castellarnau *et al*. described as key determinants of the metabolic phenotype of HCT 116 cells. The partial data set was used to apply pFBA, GIMME, ^13^C MFA and p^13^CMFA in the framework of the metabolic network defined by Tarrado-Castellarnau *et al*. [14] (S2 Zip). p^13^CMFA was applied both with and without integrating gene expression data (p^13^CMFA+ge and p^13^CMFA-ge, respectively). Two complementary metrics, Pearson’s correlation and Euclidian distance, were used to evaluate the similarity between the predicted flux distributions and the flux maps estimated by Tarrado-Castellarnau *et al*. using the full dataset[14] (Fig 4, S2 Table). The results show that p^13^CMFA-ge yields a significantly more accurate flux prediction than both pFBA (i.e., flux minimization without integrating ^13^C data), and ^13^C MFA. Interestingly, while integrating gene expression significantly enhances the accuracy of p^13^CMFA (p^13^CMFA+ge compared to p^13^CMFA-ge), such effect is less marked than the effect of adding gene expression to standard flux minimization (GIMME compared to pFBA). This is due to the fact that p^13^CMFA-ge flux predictions have already a remarkable level of accuracy; hence, less information can be gained by adding transcriptomics data. Nevertheless, even if GIMME achieves flux predictions of similar accuracy to p^13^CMFA-ge, p^13^CMFA+ge results on flux predictions that are significantly more accurate than those obtained with GIMME. Hence, in instances were only a limited number of ^13^C measurements are available, p^13^CMFA is a valid method for obtaining accurate flux estimations, regardless of the availability of gene expression data.

## Discussion

^13^C MFA is a well-established technique and has proven to be an extremely valuable tool in quantifying metabolic fluxes[9–18]. However, to fully determine fluxes through a large metabolic network, parallel labeling experiments must be performed and ^13^C propagation must be quantified in many metabolites in the network[19]. Indeed, when applying ^13^C MFA either with a small set of experimental data or with a large metabolic network, part of the ^13^C MFA solution space can be too wide to draw meaningful conclusions about the underlying flux distribution. This solution space can be reduced by removing degrees of freedom from the system, for instance, by removing reactions from the network or making reactions irreversible. However, this can introduce an arbitrary bias in the resulting flux distribution.

Here we describe p^13^CMFA, a new approach for ^13^C data integration which can overcome these limitations of ^13^C MFA and estimate a realistic solution within an undetermined ^13^C MFA solution space. This solution will be the flux distribution within the ^13^C MFA solution space that minimizes the weighted sum of reaction fluxes. Thus, it will be the most enzymatically efficient solution. In that regard, p^13^CMFA is partially based on a similar principle as pathway activity analysis (i.e., the assumption that specific fractions of isotopologues are primarily generated through the simplest combinations of pathways). However, unlike pathway activity analysis, p^13^CMFA is able to integrate all quantified isotopologue fractions and flux measurements (e.g. rates of metabolite uptake and secretion) to generate network-wide flux maps consistent with such data. Furthermore, p^13^CMFA is highly flexible; for instance, here we show that it can be used to seamlessly integrate gene expression data by giving higher weight to the minimization of the fluxes through lowly expressed enzymes.

As a proof of concept, we exemplified how p^13^CMFA can be used to estimate flux distributions integrating only limited sets of ^13^C measurements in a test case where traditional ^13^C MFA was unable to provide a narrow solution space. Furthermore, we demonstrated that, when a limited set of measurements are integrated, p^13^CMFA can yield more accurate flux predictions than both ^13^C MFA and GIMME.

p^13^C MFA does not aim to be a replacement of ^13^C MFA; instead, it seeks to supplement it by identifying the more straightforward solution in parts of the network that cannot be uniquely determined. In that regard, it can be used to quantitatively study flux distributions in instances where not enough information can be obtained with conventional ^13^C MFA. Nor does it aim to replace the direct interpretation of ^13^C data. The latter is still a suitable technique when the goal of the analysis is to compare the relative activity of well-established pathways across conditions or quantify substrate contributions rather than to generate complete flux maps.

^13^C data has been widely used to assist in drug discovery. In this regard, tracer analysis coupled with regression and correlation analyses is frequently used to characterize drug response [26–29]. Such approach uses regression and correlation statistics with binary, numeric and visual analysis to integrate drug dosage, time points, as well as all necessary biological variables in order to diagnose disturbed stable isotope labeled matrices[29]. p^13^CMFA could further expand the role of ^13^C in drug discovery by allowing the integration of ^13^C and transcriptomic data in the framework of genome-scale metabolic models. In the framework of such models, drug targets are identified by systematically simulating the effect of reactions or genes knock out to cell function[34]. This is usually attained by applying the ROOM[35] or MOMA[36] algorithms, which take a unique flux solution as input (wild-type flux distribution) to predict the most likely effect of a gene KO. Hence, p^13^CMFA results could be potentially used as ROOM/MOMA inputs allowing to take full advantage of the flux information derived from both ^13^C and transcriptomics data to predict new drug targets. With atom mappings now available on a genome-scale[37], the main obstacle to applying p^13^CMFA at a genome-scale is the high computational complexity of solving the resulting non-linear problem which increases with the size of the network. Hence, the next challenge for p^13^CMFA will be optimizing its implementation for genome-scale networks.

## Methods

### Flux spectrum

The flux spectrum[38] (i.e., the feasible range of fluxes for a given set of stoichiometric, thermodynamic and flux boundary constraints) was determined using flux variability analysis [8]. Under this approach, each flux is minimized (Eq 3) and maximized (Eq 4) subject to constraints to find the minimum (${vmin}_{i}^{FS}$) and maximum (${vmax}_{i}^{FS}$) feasible values for each flux:

$${vmin}_{i}^{FS}=\min\;v_{i}$$ (Eq 3)

subjecttoS.v=0,lb≤v≤ub

$${vmax}_{i}^{FS}=\max\;v_{i}$$ (Eq 4)

subjecttoS.v=0,lb≤v≤ub

### ^13^C MFA confidence intervals

The ^13^C MFA solution space is estimated by computing the confidence intervals for each flux. Such intervals are obtained by minimizing (Eq 5) and maximizing (Eq 6) each flux subject to constraints[5].

$${vmin}_{i}=\min\;v_{i}$$ (Eq 5)

$$subject\;to\;S.v=0,\;lb\leq v\leq ub,\;\sum_{j}{\left(\frac{E_{j}-Y_{j}(v)}{\sigma_{j}}\right)}^{2}\leq X_{opt}+T$$

$${vmax}_{i}=\max\;v_{i}$$ (Eq 6)

$$subject\;to\;S.v=0,\;lb\leq v\leq ub,\;\sum_{j}{\left(\frac{E_{j}-Y_{j}(v)}{\sigma_{j}}\right)}^{2}\leq X_{opt}+T$$

where,

*vmin*_*i*_: is the lower bound of the confidence interval for flux *i* with tolerance T;

*vmax*_*i*_: is the upper bound of the confidence interval for flux *i* with tolerance T;

Provided that the same primary objective tolerance (T) is used in computing both the p^13^CMFA solution and the ^13^C MFA confidence intervals, the p^13^CMFA solution will always fall within the boundaries of ^13^C MFA confidence intervals (*vmin*_*i*_≤*v*_*i*_≤*vmax*_*i*_).

### GIMME and pFBA

To apply GIMME and pFBA, the sum of fluxes is minimized subject only to network stoichiometry and flux boundaries (Eq 7).

$$\min\;\sum_{i}|v_{i}|\cdot w_{i}$$ (Eq 7)

subjecttoS.v=0,lb≤v≤ub

In GIMME, flux minimization weights are derived from gene expression measurements, whereas in pFBA all reactions are given the same minimization weight[20,22].

### Transcriptomic analysis

Transcriptomic data of HUVECs and HCT 116 cells published by Weigand *et al*.[32] and Tarrado-Castellarnau[14] *et al*., respectively, were obtained from the Gene Expression Omnibus repository[39]. A Robust Multichip Analysis gene-level normalization[40] was performed with the Oligo package for R[41].

Using gene-protein-reaction rules, normalized transcript intensities were mapped to each enzyme-catalyzed reaction or protein-facilitated transport process. The weight given to the minimization of fluxes was assigned according to the following equation:

$$w_{i}=1+\max\left(Th-{ge}_{i},0\right)$$ (Eq 8)

where,

*ge*_*i*_ is the gene expression value assigned to reaction *i*;

*Th* is the gene expression threshold. Fluxes through reactions with gene expression levels below this threshold are given additional minimization weight;

Using the same criteria as GIM^3^E[22], Th was set at the maximum gene expression value found in the set of genes mapped to the metabolic network (Eq 9):

Th=max(ge) (Eq 9)

Using this threshold, the information gained from integrating available gene expression measurements is maximized. Other Th values were tested in the validation case study[14] and using the maximum gene expression as the threshold was found to yield the most accurate flux predictions (S3 Table).

### p^13^CMFA implementation

p^13^CMFA was implemented in Iso2Flux, our in-house developed ^13^C MFA software (https://github.com/cfoguet/iso2flux/releases/tag/0.7.2).

Iso2Flux computes steady-state flux distributions as the product of the null space of the stoichiometric matrix and the vector of free fluxes. Reversible reactions are split into forward and reverse reactions. For each reversible reaction, a turnover variable (*t*_*i*_) is introduced defining the flux that is common to the forward (*v*_*i*_^*f*^) and reverse (*v*_*i*_^*r*^) reactions. These variables are used to assign values to the fluxes of the forward and reverse reactions as a function of the steady-state net flux (v_i_).

$$v_{i}^{f}=t_{i}+\max\left(v_{i},0\right)$$ (Eq 10)

$$v_{i}^{r}=t_{i}-\min\left(v_{i},0\right)$$ (Eq 11)

Iso2flux uses the Elementary Metabolite Unit (EMU) framework[1] to build the ^13^C propagation model. This framework is based on a highly efficient decomposition method that identifies the minimum amount of isotopologue transitions required to simulate the experimentally quantified isotopologues according to the defined carbon propagation rules. The isotopologue transitions are grouped into decoupled systems based on isotopologue size. Balance equations are built around each isotopologue fraction under the assumption of isotopic steady state (S1 Fig). Using the steady-state flux distribution as an input, systems of equations around isotopologues balances are solved sequentially starting with the smallest isotopologue size [1] using the fsolve function of the SciPy library (https://scipy.org/scipylib/index.html). Solving such system predicts the isotopologue distribution associated with a given steady-state flux distribution (*Y*_*j*_*(v))*.

The self-adaptive differential evolution (SADE) algorithm from PyGMO (Python Parallel Global Multiobjective Optimizer, https://github.com/esa/pagmo2) was used to find the optimal solution of the ^13^C MFA (Eq 1) and p^13^CMFA (Eq 2) problems. SADE was parallelized using the generalized island-model paradigm. Under such implementation, SADE is run in parallel in different CPU processes (islands). After a given number of SADE iterations (generations), the best solutions (individuals) in each SADE process (island) are shared to parallel SADE processes (migrate to adjacent islands). To prevent bias from the starting solutions (starting populations), the islands are seeded through random sampling of all variables. Free fluxes variables are sampled using the optGpSampler implemented into COBRApy[42,43]. Turnover variables are sampled using the random.uniform function built into python. The algorithm was run with 7 islands, each with a population of 60, and with migrations between islands set to occur every 400 generations. For the analyzed ^13^C MFA and p^13^CMFA problems, repeated iterations of the algorithm were shown to reliably converge towards the same minimal objective function value.

### Accommodating large metabolite pools

At the beginning of a ^13^C experiment, all internal metabolites are unlabeled (m0). Progressively, these products are enriched in ^13^C, with the subsequent decrease in m0. Isotopic steady state is quickly reached for small pools of metabolites but not necessarily for larger pools such as those of fatty acids, glycogen or metabolites present in large concentrations in the external medium[44]. For these larger pools, unlabeled isotopologues m0 are oversized and might not quickly decrease to the theoretical value that should be reached at steady-state.

However, it is possible to represent the effect of large pools in the framework of steady-state ^13^C MFA through the addition of a virtual reaction. This reaction replaces labeled isotopologues by unlabeled isotopologues in metabolites with large pools. With p^13^CMFA, the flux through this virtual reaction can be minimized. Effectively, this allows correcting steady-state ^13^C simulations for large pools while identifying the solutions that require the minimum amount of correction.

### Evaluating the significance of the difference between correlation coefficients

The statistical significance of the difference between correlation coefficients was evaluated using the Fisher r-to-z transformation[33]. Following this approach, Pearson’s correlation coefficients (r) can be converted to a z-score (r’):

$$r^{\prime }=\frac{1}{2}\cdot Ln\left(\frac{1+r}{1-r}\right)$$ (Eq 12)

The variance of z (Sz) will depend only on the sample size (n):

$$S_{z}=\sqrt{\frac{1}{n-3}}$$ (Eq 13)

From Eq 12 and Eq 13, the significance of the difference between two correlation coefficients (r1 and r2) can be evaluated by computing the z score corresponding to such difference (Eq 14) and its associated p-value.

$$Z=\frac{{r^{\prime }}_{1}-{r^{\prime }}_{2}}{\sqrt{\frac{1}{n_{r1}-3}+\frac{1}{n_{r2}-3}}}$$ (Eq 14)

### Experimental methods

Human Umbilical Vein Endothelial Cells (HUVECs-pooled, Lonza) were maintained on 1% gelatin-coated flasks at 37°C in a humidified atmosphere of 5% CO_2_ and 95% air in MCDB131 (Gibco) medium, supplemented with the recommended quantity of endothelial growth medium (EGM) SingleQuots (Lonza), 10% fetal bovine serum (FBS) (Gibco), 2 mM glutamine (Gibco) and 0.1% Streptomycin (100 μg/mL)/Penicillin (100 units/mL) (S/P) (Gibco). 1 × 10^6^ HUVECs were seeded in 1% gelatin-coated cell culture plates for 6h, and then the maintenance medium was replaced with the MCDB131 basal medium, supplemented with 2% FBS, 2 mM glutamine and 0.1% S/P and cells were incubated overnight for nutrient deprivation. After nutrient deprivation, the medium was replaced with a restricted medium containing MCDB131 medium supplemented with 2% FBS, 2 mM glutamine and 0.1% S/P with 10 mM of 50% [1,2-^13^C_2_]-glucose (Sigma-Aldrich) and cells were incubated for 40h in a humidified atmosphere with 5% CO_2_ and 1% O_2_ at 37°C. Both at the beginning (t = 0h) and the end (t = 40h) of incubation, media and pellets were collected. On the one hand, media and cell pellets were used for analyzing isotopologue abundances for glucose, lactate, glutamate, RNA ribose and glycogen. Raw data are publicly available in the MetaboLights repository at http://www.ebi.ac.uk/metabolights [31], with accession number MTBLS412. Isolation, derivatization and analysis details are described in MetaboLights. Glucose, lactate, glutamate, and glutamine concentrations were determined in media samples for estimation of secretion or uptake rates of these metabolites using spectrophotometric methods[45]. Also, the net rate of glycogen re-utilization into glucose was estimated by quantifying glycogen content at initial and final time points using [U-^13^C-D_7_]-glucose as recovery standard[46]. All biochemical data were normalized by cell number, and by incubation time (h). The resulting rates–expressed in micromoles of metabolite consumed/produced/transformed per hour per million cells (μmol·h^-1^·million-cells^-1^)–were 0.463, 0.099, 0.050 and 1.169 for glucose uptake, glutamine uptake, glutamate secretion, and lactate secretion, respectively, and a net transformation of glycogen of 0.000175.

## Supporting information

S1 Fig Example of isotopologue balance equations in a toy metabolic network. In this toy metabolic network, two mono-carbon metabolites (C_a_ and C_b_) are condensed into a bi-carbon metabolite (C_a_-C_b_) through a reaction with a flux v_1_. Metabolite C_a_-C_b_ is removed from the system at a rate of v_2_. For each metabolite, isotopologue fractions (M_x_) are defined as the relative abundance of the metabolite with *x* number of ^13^C substitutions. Isotopologue balances for metabolite C_a_-C_b_ are indicated. Under the assumption of isotopic steady state (i.e., isotopologue fractions are constant in time) and given v_1_ and v_2_, and a set of isotopologue fractions for C_a_ and C_b_ (assumed a constant input), the system can be solved to identify the steady-state isotopologue fractions for metabolite C_a_-C_b_.
(TIF)

S1 Table Flux spectrum, GIMME, ^13^C MFA and p^13^CMFA flux solutions for all net reaction fluxes in the HUVECs case study. Fluxes are expressed in μmol·h^-1^·million-cells^-1^.
(XLSX)

S2 Table Comparison between the reference flux map in HCT 116 cells and the flux maps computed from the partial data set using ^13^C MFA, pFBA, GIMME, and p^13^CMFA. Fluxes are indicated in μmol·h^-1^·million-cells^-1^.
(XLSX)

S3 Table Comparison between the reference flux map in HCT 116 cells and the flux maps computed from the partial data set with p^13^CMFA using different gene expression percentiles as thresholds for adding additional weight to flux minimization. Fluxes are indicated in μmol·h^-1^·million-cells^-1^.
(XLSX)

S1 ZIP Files describing the metabolic network, carbon propagation rules, and experimental data used for the HUVECs case study. The files are inputs for running p^13^CMFA on Iso2Flux.
(ZIP)

S2 ZIP Files describing the metabolic network, carbon propagation rules, and experimental data used for the HCT 116 cells case study. The files are inputs for running p^13^CMFA on Iso2Flux.
(ZIP)

## References

[1] Antoniewicz MR, Kelleher JK, Stephanopoulos G. Elementary metabolite units (EMU): a novel framework for modeling isotopic distributions. Metab Eng. 2007;9: 68–86. 10.1016/j.ymben.2006.09.001 17088092 PMC1994654

[2] Buescher JM, Antoniewicz MR, Boros LG, Burgess SC, Brunengraber H, Clish CB, et al A roadmap for interpreting (13)C metabolite labeling patterns from cells. Curr Opin Biotechnol. 2015;34: 189–201. 10.1016/j.copbio.2015.02.003 25731751 PMC4552607

[3] Zamboni N, Saghatelian A, Patti GJ. Defining the metabolome: size, flux, and regulation. Mol Cell. 2015;58: 699–706. 10.1016/j.molcel.2015.04.021 26000853 PMC4831058

[4] Orth JD, Thiele I, Palsson BØ. What is flux balance analysis? Nat Biotechnol. 2010;28: 245–8. 10.1038/nbt.1614 20212490 PMC3108565

[5] Antoniewicz MR, Kelleher JK, Stephanopoulos G. Determination of confidence intervals of metabolic fluxes estimated from stable isotope measurements. Metab Eng. 2006;8: 324–337. 10.1016/j.ymben.2006.01.004 16631402

[6] Niedenführ S, Wiechert W, Nöh K. How to measure metabolic fluxes: A taxonomic guide for 13C fluxomics. Curr Opin Biotechnol. 2015;34: 82–90. 10.1016/j.copbio.2014.12.003 25531408

[7] Balcells C, Foguet C, Tarragó-Celada J, de Atauri P, Marin S, Cascante M. Tracing metabolic fluxes using mass spectrometry: Stable isotope-resolved metabolomics in health and disease. TrAC Trends Anal Chem. 2019; 10.1016/j.trac.2018.12.025

[8] Gudmundsson S, Thiele I. Computationally efficient flux variability analysis. BMC Bioinformatics. 2010;11: 489 10.1186/1471-2105-11-489 20920235 PMC2963619

[9] Niklas J, Sandig V, Heinzle E. Metabolite channeling and compartmentation in the human cell line AGE1.HN determined by ^13^C labeling experiments and13C metabolic flux analysis. J Biosci Bioeng. 2011;112: 616–623. 10.1016/j.jbiosc.2011.07.021 21865082

[10] Walther JL, Metallo CM, Zhang J, Stephanopoulos G. Optimization of13C isotopic tracers for metabolic flux analysis in mammalian cells. Metab Eng. 2012;14: 162–171. 10.1016/j.ymben.2011.12.004 22198197 PMC3586220

[11] Metallo CM, Gameiro PA, Bell EL, Mattaini KR, Yang J, Hiller K, et al Reductive glutamine metabolism by IDH1 mediates lipogenesis under hypoxia. Nature. 2012;481: 380–384. 10.1038/nature10602 22101433 PMC3710581

[12] Grassian AR, Parker SJ, Davidson SM, Divakaruni AS, Green CR, Zhang X, et al IDH1 mutations alter citric acid cycle metabolism and increase dependence on oxidative mitochondrial metabolism. Cancer Res. 2014;74: 3317–3331. 10.1158/0008-5472.CAN-14-0772-T 24755473 PMC4885639

[13] Crown SB, Kelleher JK, Rouf R, Muoio DM, Antoniewicz MR. Comprehensive metabolic modeling of multiple 13C-isotopomer data sets to study metabolism in perfused working hearts. Am J Physiol Heart Circ Physiol. 2016;311: H881–H891. 10.1152/ajpheart.00428.2016 27496880 PMC5114467

[14] Tarrado‐Castellarnau M, de Atauri P, Tarragó‐Celada J, Perarnau J, Yuneva M, Thomson TM, et al De novo MYC addiction as an adaptive response of cancer cells to CDK4/6 inhibition. Mol Syst Biol. 2017;13: 940 10.15252/msb.20167321 28978620 PMC5658703

[15] Carinhas N, Koshkin A, Pais DAM, Alves PM, Teixeira AP. 13 C-metabolic flux analysis of human adenovirus infection: Implications for viral vector production. Biotechnol Bioeng. 2017;114: 195–207. 10.1002/bit.26063 27477740

[16] DeWaal D, Nogueira V, Terry AR, Patra KC, Jeon S-M, Guzman G, et al Hexokinase-2 depletion inhibits glycolysis and induces oxidative phosphorylation in hepatocellular carcinoma and sensitizes to metformin. Nat Commun. 2018;9: 446 10.1038/s41467-017-02733-4 29386513 PMC5792493

[17] Gopalakrishnan S, Maranas CD. ^13^C metabolic flux analysis at a genome-scale. Metab Eng. 2015;32: 12–22. 10.1016/j.ymben.2015.08.006 26358840

[18] Foguet C, Marin S, Selivanov VA, Fanchon E, Lee W-NP, Guinovart JJ, et al HepatoDyn: A Dynamic Model of Hepatocyte Metabolism That Integrates 13C Isotopomer Data. Lewis NE, editor. PLoS Comput Biol. 2016;12: e1004899 10.1371/journal.pcbi.1004899 27124774 PMC4849781

[19] Crown SB, Long CP, Antoniewicz MR. Optimal tracers for parallel labeling experiments and 13C metabolic flux analysis: A new precision and synergy scoring system. Metab Eng. 2016;38: 10–18. 10.1016/j.ymben.2016.06.001 27267409 PMC5891732

[20] Lewis NE, Hixson KK, Conrad TM, Lerman JA, Charusanti P, Polpitiya AD, et al Omic data from evolved E. coli are consistent with computed optimal growth from genome-scale models. Mol Syst Biol. 2010;6: 390 10.1038/msb.2010.47 20664636 PMC2925526

[21] Becker SA, Palsson BO. Context-Specific Metabolic Networks Are Consistent with Experiments. Sauro HM, editor. PLoS Comput Biol. 2008;4: e1000082 10.1371/journal.pcbi.1000082 18483554 PMC2366062

[22] Schmidt BJ, Ebrahim A, Metz TO, Adkins JN, Palsson BØ, Hyduke DR. GIM3E: condition-specific models of cellular metabolism developed from metabolomics and expression data. Bioinformatics. 2013;29: 2900–8. 10.1093/bioinformatics/btt493 23975765 PMC3810847

[23] Antoniewicz MR. A guide to 13C metabolic flux analysis for the cancer biologist. Exp Mol Med. 2018;50: 19 10.1038/s12276-018-0060-y 29657327 PMC5938039

[24] Dong W, Keibler MA, Stephanopoulos G. Review of metabolic pathways activated in cancer cells as determined through isotopic labeling and network analysis. Metabolic Engineering. 2017 pp. 113–124. 10.1016/j.ymben.2017.02.002 28192215 PMC5552450

[25] Bruntz RC, Lane AN, Higashi RM, Fan TWM. Exploring cancer metabolism using Stable isotope-resolved metabolomics (SIRM). J Biol Chem. 2017;292: 11601–11609. 10.1074/jbc.R117.776054 28592486 PMC5512057

[26] Harrigan GG, Colca J, Szalma S, Boros LG. PNU-91325 increases fatty acid synthesis from glucose and mitochondrial long chain fatty acid degradation: A comparative tracer-based metabolomics study with rosiglitazone and pioglitazone in HepG2 cells. Metabolomics. 2006;2: 21–29. 10.1007/s11306-006-0015-5 24489530 PMC3906712

[27] Beger RD, Hansen DK, Schnackenberg LK, Cross BM, Fatollahi JJ, Lagunero FT, et al Single valproic acid treatment inhibits glycogen and RNA ribose turnover while disrupting glucose-derived cholesterol synthesis in liver as revealed by the [U-13C6]-d-glucose tracer in mice. Metabolomics. 2009;5: 336–345. 10.1007/s11306-009-0159-1 19718458 PMC2731156

[28] Cantoria MJ, Boros LG, Meuillet EJ. Contextual inhibition of fatty acid synthesis by metformin involves glucose-derived acetyl-CoA and cholesterol in pancreatic tumor cells. Metabolomics. 2014;10: 91–104. 10.1007/s11306-013-0555-4 24482631 PMC3890070

[29] Boros LG, Beger RD, Meuillet EJ, Colca JR, Szalma S, Thompson PA, et al Targeted 13C-Labeled Tracer Fate Associations for Drug Efficacy Testing in Cancer Tumor Cell Metabolism. Vienna: Springer Vienna; 2015 pp. 349–372. 10.1007/978-3-7091-1824-5_15

[30] Varma V, Boros LG, Nolen GT, Chang CW, Wabitsch M, Beger RD, et al Metabolic fate of fructose in human adipocytes: a targeted ^13^C tracer fate association study. Metabolomics. 2015;11: 529–544. 10.1007/s11306-014-0716-0 25972768 PMC4419153

[31] Haug K, Salek RM, Conesa P, Hastings J, de Matos P, Rijnbeek M, et al MetaboLights—an open-access general-purpose repository for metabolomics studies and associated meta-data. Nucleic Acids Res. 2013;41: D781–6. 10.1093/nar/gks1004 23109552 PMC3531110

[32] Weigand JE, Boeckel J-N, Gellert P, Dimmeler S. Hypoxia-Induced Alternative Splicing in Endothelial Cells. Preiss T, editor. PLoS One. 2012;7: e42697 10.1371/journal.pone.0042697 22876330 PMC3411717

[33] Weaver B, Wuensch KL. SPSS and SAS programs for comparing Pearson correlations and OLS regression coefficients. Behav Res Methods. 2013;45: 880–895. 10.3758/s13428-012-0289-7 23344734

[34] de Mas IM, Aguilar E, Jayaraman A, Polat IH, Martín-Bernabé A, Bharat R, et al Cancer cell metabolism as new targets for novel designed therapies. Future Med Chem. 2014;6: 1791–1810. 10.4155/fmc.14.119 25574531

[35] Shlomi T, Berkman O, Ruppin E. Regulatory on/off minimization of metabolic flux changes after genetic perturbations. Proc Natl Acad Sci U S A. 2005;102: 7695–700. 10.1073/pnas.0406346102 15897462 PMC1140402

[36] Segre D, Vitkup D, Church GM. Analysis of optimality in natural and perturbed metabolic networks. Proc Natl Acad Sci. 2002;99: 15112–15117. 10.1073/pnas.232349399 12415116 PMC137552

[37] Brunk E, Sahoo S, Zielinski DC, Altunkaya A, Dräger A, Mih N, et al Recon3D enables a three-dimensional view of gene variation in human metabolism. Nat Biotechnol. 2018;36: 272–281. 10.1038/nbt.4072 29457794 PMC5840010

[38] Llaneras F, Picó J. An interval approach for dealing with flux distributions and elementary modes activity patterns. J Theor Biol. 2007;246: 290–308. 10.1016/j.jtbi.2006.12.029 17292923

[39] Edgar R. Gene Expression Omnibus: NCBI gene expression and hybridization array data repository. Nucleic Acids Res. 2002;30: 207–210. 10.1093/nar/30.1.207 11752295 PMC99122

[40] Irizarry RA, Hobbs B, Collin F, Beazer-Barclay YD, Antonellis KJ, Scherf U, et al Exploration, normalization, and summaries of high density oligonucleotide array probe level data. Biostatistics. 2003;4: 249–64. 10.1093/biostatistics/4.2.249 12925520

[41] Carvalho BS, Irizarry RA. A framework for oligonucleotide microarray preprocessing. Bioinformatics. 2010;26: 2363–2367. 10.1093/bioinformatics/btq431 20688976 PMC2944196

[42] Ebrahim A, Lerman JA, Palsson BO, Hyduke DR. COBRApy: COnstraints-Based Reconstruction and Analysis for Python. BMC Syst Biol. 2013;7: 74 10.1186/1752-0509-7-74 23927696 PMC3751080

[43] Megchelenbrink W, Huynen M, Marchiori E. optGpSampler: An Improved Tool for Uniformly Sampling the Solution-Space of Genome-Scale Metabolic Networks. Rogers S, editor. PLoS One. 2014;9: e86587 10.1371/journal.pone.0086587 24551039 PMC3925089

[44] Selivanov VA, Meshalkina LE, Solovjeva ON, Kuchel PW, Ramos-Montoya A, Kochetov GA, et al Rapid simulation and analysis of isotopomer distributions using constraints based on enzyme mechanisms: an example from HT29 cancer cells. Bioinformatics. 2005;21: 3558–64. 10.1093/bioinformatics/bti573 16002431

[45] Benito A, Polat IH, Noé V, Ciudad CJ, Marin S, Cascante M. Glucose-6-phosphate dehydrogenase and transketolase modulate breast cancer cell metabolic reprogramming and correlate with poor patient outcome. Oncotarget. 2017;8: 106693–106706. 10.18632/oncotarget.21601 29290982 PMC5739767

[46] Vizán P, Sánchez-Tena S, Alcarraz-Vizán G, Soler M, Messeguer R, Pujol MD, et al Characterization of the metabolic changes underlying growth factor angiogenic activation: identification of new potential therapeutic targets. Carcinogenesis. 2009;30: 946–52. 10.1093/carcin/bgp083 19369582

