# Supplementary figures and images for: p13CMFA: Parsimonious 13C metabolic flux analysis
Source: PLoS Comput Biol. 2019 Sep 6;15(9):e1007310. doi: 10.1371/journal.pcbi.1007310 (PMC6750616; doi:10.1371/journal.pcbi.1007310)

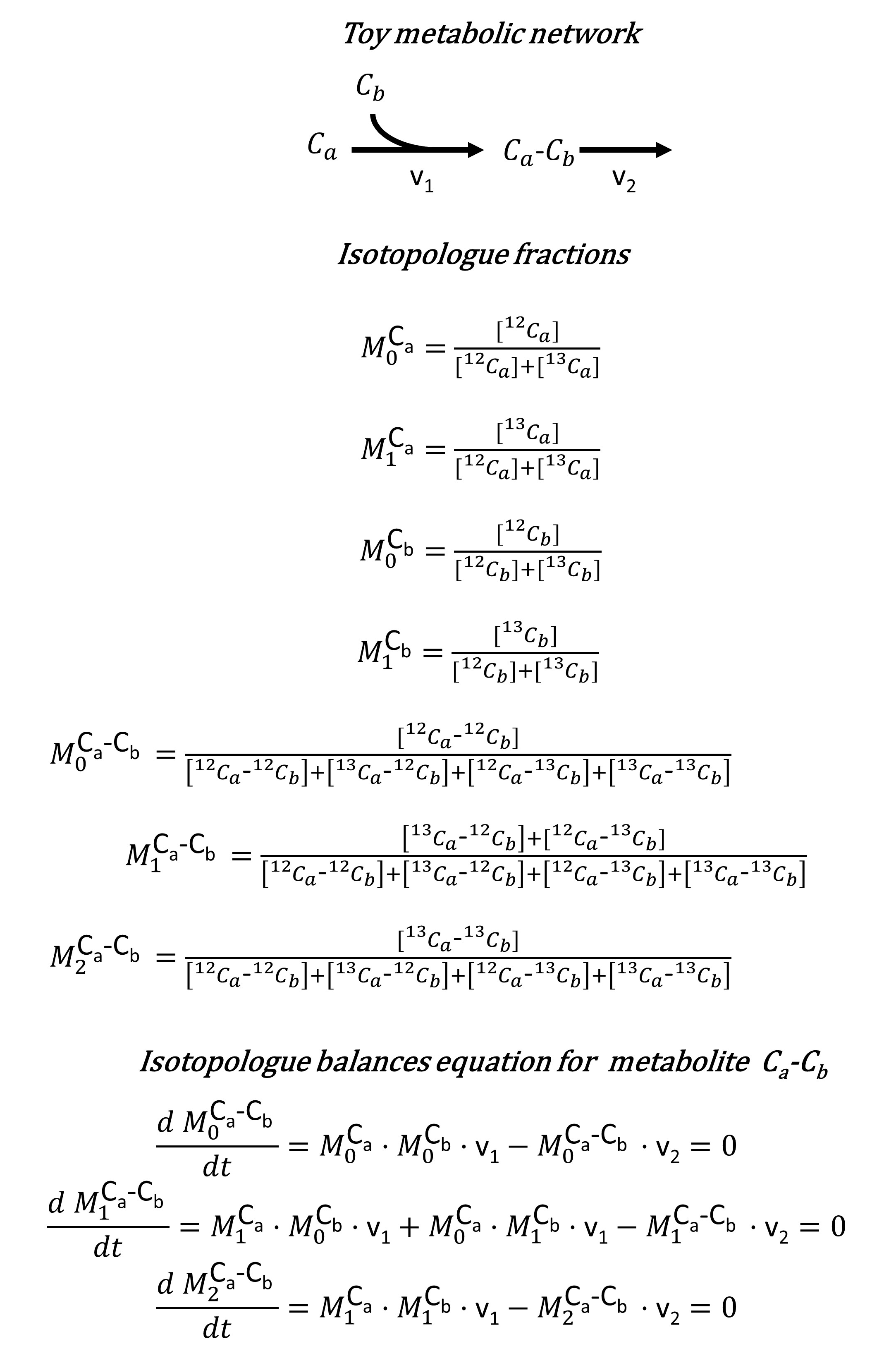

Supplement: S1 Fig — In this toy metabolic network, two mono-carbon metabolites (Ca and Cb) are condensed into a bi-carbon metabolite (Ca-Cb) through a reaction with a flux v1. Metabolite Ca-Cb is removed from the system at a rate of v2. For each metabolite, isotopologue fractions (Mx) are defined as the relative abundance of the metabolite with x number of 13C substitutions. Isotopologue balances for metabolite Ca-Cb are indicated. Under the assumption of isotopic steady state (i.e., isotopologue fractions are constant in time) and given v1 and v2, and a set of isotopologue fractions for Ca and Cb (assumed a constant input), the system can be solved to identify the steady-state isotopologue fractions for metabolite Ca-Cb. (TIF) [file pcbi.1007310.s001.tif]
